# p53 Status Correlates with the Risk of Recurrence in Non-Muscle Invasive Bladder Cancers Treated with Bacillus Calmette–Guérin: A Meta-Analysis

**DOI:** 10.1371/journal.pone.0119476

**Published:** 2015-03-05

**Authors:** Xiaofeng Zhou, Guan Zhang, Ye Tian

**Affiliations:** 1 Department of Urology, Beijing Friendship Hospital, Capital Medical University, Yongan Road 95, Beijing 100050, China; 2 Department of Urology, China-Japan Friendship Hospital, Yinghua East Road 2, Beijing 100029, China; Okayama University, JAPAN

## Abstract

**Objective:**

Published studies have yielded inconsistent results on the relationship between p53 status and the prognosis of non-muscle invasive bladder cancer (NMIBC) treated with Bacillus Calmette–Guérin (BCG) intravesical therapy. Therefore, we performed a meta-analysis to evaluate the prognostic value of p53 in NMIBC treated with BCG.

**Methods:**

We systematically searched for relevant literature in PubMed, EMBASE, CNKI, and Chinese Wanfang databases. Hazard ratios (HRs) with 95% confidence intervals (CIs) were combined as the effect size (ES) across studies for recurrence-free survival (RFS) and progression-free survival (PFS).

**Results:**

A total of 11 studies, consisting of 1,049 participants, met the criteria. Overall, there was no clear relationship between p53 status and RFS or PFS for NMIBC patients treated with BCG (HR: 1.40, 95% CI: 0.91-2.16; HR: 1.37, 95% CI: 0.90-2.09, respectively). Obvious heterogeneity was observed across the studies (I^2^ = 69.5%, *P* = 0.001; I^2^ = 44.7%, *P* = 0.081, respectively). In stratified analysis by region, p53 overexpression was a predictor of poor RFS in Asian populations (HR: 1.57, 95% CI: 1.08-2.27). In addition, after excluding the studies that possibly contributed to the heterogeneity by the Galbraith plot, the overall association for RFS became statistically significant (HR: 1.38 95% CI: 1.08-1.77) without evidence of heterogeneity (I^2^ = 0.0%, *P* = 0.499).

**Conclusion:**

This meta-analysis suggests that p53 overexpression in NMIBC patients treated with BCG may be associated with RFS, especially in Asian populations. Because of the heterogeneity and other limitations, further studies with rigid criteria and large populations are still warranted to confirm our findings.

## Introduction

Bladder cancer ranks ninth in the worldwide cancer incidence [1]. There were a total of 386,300 new cases and 150,200 deaths from bladder cancer occurred in 2008 [2]. Among these, approximately 70% of bladder tumors are non-muscle invasive bladder cancers (NMIBCs) at the time of presentation [3]. Recently, after initial transurethral resection of bladder tumors (TURB), adjuvant intravesical treatment with Bacillus Calmette–Guérin (BCG) was selected as the first-line therapy for patients with NMIBC. However, predicting which patients are destined to fail BCG treatment is still a very difficult task [4].

Molecular tumor markers hold considerable promise for accurately predicting the recurrence and progression of NMIBC patients treated with BCG intravesical therapy. Deregulation of the cell-cycle machinery is common in bladder cancer, involving alterations in various proteins such as cyclin D1, Rb, p16, p21, p27, and p53 [5,6]. The value of pretreatment p53 overexpression on the prognosis of NMIBC patients treated with BCG has been widely studied and discussed. As a result, most of reports are in consensus that p53 is not associated with the prognosis [7–11]. However, several studies have suggested different results. For example, three independent studies reported a positive association between strong p53 overexpression and the risk of recurrence in NMIBC patients treated with intravesical BCG [12–14], whereas Oderda et al.’ study suggested a negative association between them [15]. In addition, studies conducted by Lopez-Beltran et al. and Lacombe et al. indicated that increased p53 expression was associated with the risk of progression [16,17].

Meta-analysis is a useful method for overcoming the small sample size problem of individual studies and enhancing the low statistical power [18,19]. Considering the inconsistent results of published articles, we performed a comprehensive meta-analysis of all published studies to determine the value of p53 as a prognostic marker for NMIBC patients treated with BCG intravesical instillations.

## Methods

### Search strategy

This systematic review and meta-analysis was performed according to the Preferred Reporting Items for Systematic Reviews and Meta-Analyses (PRISMA) statement [20] (S1 PRISMA Checklist). Published articles studying the prognostic value of p53 in NMIBC treated with BCG were identified from PubMed, EMBASE, CNKI, and Chinese Wanfang databases with the following search terms: “bladder cancer or bladder carcinoma or bladder neoplasm or bladder tumor”, “Bacillus Calmette–Guérin or BCG”, and “p53 or TP53”. The final search was conducted on November 30, 2014. The references of the retrieved articles and reviews were also screened for additional relevant studies. The eligible publications were selected by two reviewers, and controversial articles were adjudicated by a third reviewer.

### Inclusion criteria

The following criteria were applied to identify the eligible articles: (a) to evaluate the relationship between p53 status and the prognosis of NMIBC patients treated with BCG; (b) to detect p53 status in the primary tumor tissues using immunohistochemistry (IHC); (c) to provide adequate data to estimate the hazard ratios (HRs) and their 95% confidence intervals (CIs); and (d) publications in English or Chinese. For overlapping studies, the most recent and detailed study was eligible for inclusion in this meta-analysis.

### Data extraction

According to the inclusion criteria listed above, two reviewers independently extracted the following information: the first author’s surname, publication year, country in which the study was conducted, number of patients analyzed, age of the patients, follow-up time, disease stage, cut-off value, and prognostic data. Disagreement was resolved by a consensus discussion among all authors.

### Statistical methods

The impact of p53 expression on survival was quantified with the combined HRs and 95% CIs. The HR and 95% CI of each study were directly extracted from each publication. If not directly available, they were calculated from the available data in the articles using methods described by Parmar et al. [21]. In this meta-analysis, the DerSimonian–Laird random-effect model [22] was applied, which considered both within-study and between-study variation. By convention, an HR > 1 corresponded to a poor outcome for increased p53, while HR < 1 indicated a favorable prognosis. The impact of increased p53 expression on survival was considered statistically significant if the 95% CI did not exceed 1. Subgroup analyses by different analytical methods (region, sample size, follow-up time, stage, cut-off, publication year, and patient age) were performed. We also performed a cumulative meta-analysis by sorting the studies according to the publication year [23].

Statistical heterogeneity across studies was assessed by the Chi-square-based Q test and I^2^ statistic [24]. A *P* value < 0.10 was considered statistically significant. The value of I^2^ was used to evaluate the degree of heterogeneity (no heterogeneity: I^2^ < 25%; moderate heterogeneity: I^2^ = 25–50%; and large or extreme heterogeneity: I^2^ > 50%). In addition, the Galbraith plot was used to detect the possible sources of heterogeneity [25], and a re-analysis was conducted after excluding the studies that possibly contributed to the heterogeneity. Additionally, residual maximum likelihood (REML)-based random-effects meta-regression analysis was used to explore the potential sources of heterogeneity [26,27].

### Estimation of the publication bias

The potential publication bias was estimated using Begg’s funnel plot [28], Egger’s test [29], and the trim-and-fill method [30]. *P* < 0.05 was considered representative of a statistically significant publication bias. All of the statistical analyses were performed with STATA 11.0 (StataCorp, College Station, Texas USA) using two-sided *P* values.

## Results

### Study selection

**Fig 1:**
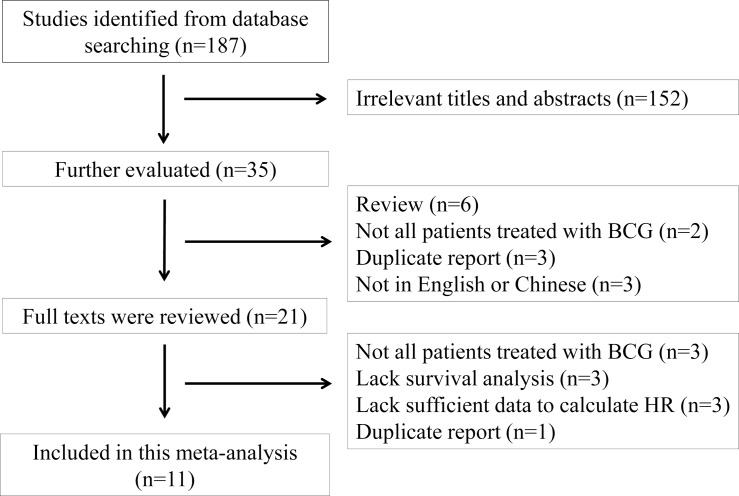
Flowchart of study selection.

As shown in Fig. 1, a total of 187 articles were identified using the search strategy as described above. After carefully reading the titles and abstracts, 166 studies that were not relevant to our aim were excluded. Upon further review of the remaining 21 articles, 3 were excluded because not all of the included patients were treated with BCG, 3 were excluded because they did not provide sufficient data to calculate the HR, 3 were excluded because they did not include survival analysis, and 1 was excluded because it had data that overlapped with other studies. After selection, a total of 11 articles were finally included in the evaluation of the prognostic value of p53 status in NMIBC patients treated with BCG.

### Characteristics of the eligible studies

The clinical features of these 11 eligible studies are summarized in Table 1. These studies were published between 1996 and 2013. Three articles evaluated patients from Korea, 2 from Italy, 2 from France, 1 from the USA, 1 from Singapore, 1 from Spain, and 1 from two countries (Netherlands and Canada). The total number of patients was 1,049, with study sample sizes ranging from 27 to 275 patients. The follow-up period was at least 24 months and the follow-up period for 5 studies was at least 60 months. The definition of the cut-off value for high p53 expression was varied and 6 studies used the percentage of stained cells not less than 20%. The HRs and 95% CIs were recorded for each study using reported data or the methods described above. Recurrence-free survival (RFS) was reported in 9 studies and progression-free survival (PFS) reported in 8 studies.

**Table 1:** Main characteristics of all studies included in this meta-analysis.

Study	Year	Country	No. of Patients	Age (years)	Follow up (months)	Stage	Cut off	Survival analysis
Oderda et al	2013	Italy	192	73.2 (SD 11.9)	100 (2–229)	All NMIBC	20%	RFS,PFS
Park et al	2013	Korea	61	66 (31–85)	60 (6–217)	T1G3	10%	RFS,PFS
van Rhijn et al	2012	Netherlands,Canada	129	68.8 (SD 9.9)	78 (39.6–110.4)	T1	10%	RFS,PFS
Oh et al	2010	Korea	275	63.9	50.4 (0–115.2)	All NMIBC	60%	RFS
Cormio et al	2009	Italy	27	69 (57–81)	60 (15–135)	T1G3	20%	RFS,PFS
Esuvaranathan et al	2007	Singapore	53	63.5	54 (6–114)	All NMIBC	Score > 3	RFS,PFS
Lopez-Beltran et al	2004	Spain	51	69.96 (49–89)	63.82 (60–144)	T1G3	6%	PFS
Saint et al	2004	France	102	64.6 (36.8–88)	40 (4.4–164.5)	All NMIBC	20%	RFS
Peyromaure et al	2002	France	29	72.2 (52–87)	36.7 (1–108)	T1G3	20%	RFS,PFS
Lee et al	1997	Korea	32	57.1 (30–81)	All > 24	T1G2–3	20%	RFS
Lacombe et al	1996	USA	98	65 (28–83)	44 (2–66)	All NMIBC	20%	PFS

No., number; NMIBC, non-muscle-invasive bladder cancer; RFS, recurrence-free survival; PFS, progression-free survival.

### Impact of p53 expression on RFS

**Fig 2:**
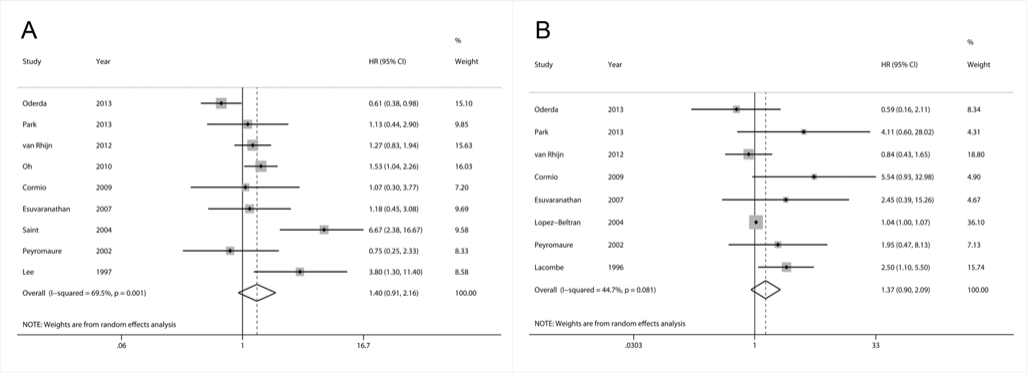
Forest plots of HRs estimated for the relationship between p53 expression and RFS (A) or PFS (B) among NMIBC patients treated with BCG.

Nine articles contained information on the correlation between p53 expression and RFS in NMIBC patients treated with BCG. The combined data from all these studies suggested that p53 overexpression was not statistically associated with RFS with a pooled HR estimate of 1.40 (95% CI: 0.91–2.16) (Fig. 2A). Obvious heterogeneity was observed (I^2^ = 69.5%, *P* = 0.001). However, subgroup analysis by region showed that increased p53 expression was significantly correlated with RFS in Asian populations (HR: 1.57, 95% CI: 1.08–2.27), and there was no significant heterogeneity across the studies (I^2^ = 10.7%, *P* = 0.339). Likewise, stratification analysis by patient age indicated that p53 was also significantly associated with RFS in patients ≤ 65 years old (HR: 2.43, 95% CI: 1.16–5.12), which had significant heterogeneity (I^2^ = 70.2%, *P* = 0.018) (Table 2)

**Table 2:** Subgroup results of RFS and heterogeneity test.

			Heterogeneity test		
Variables	Study number	HR (95% CI)	Q	*P*	I^2^ (%)
Total RFS	9	1.40 (0.91–2.16)	26.20	0.001	69.5
Region					
Asian	4	1.57 (1.08–2.27)	3.36	0.339	10.7
Caucasian	5	1.28 (0.62–2.67)	19.99	0.001	80.0
Sample size					
≥ 100	4	1.50 (0.76–2.95)	21.26	< 0.001	85.9
< 100	5	1.33 (0.78–2.24)	4.89	0.299	18.2
Follow-up (months)					
≥ 60	4	0.94 (0.61–1.46)	5.30	0.151	43.4
< 60	4	1.75 (0.81–3.75)	10.43	0.015	71.2
Stage					
All NMIBC	4	1.54 (0.67–3.52)	21.26	< 0.001	85.9
T1G3	3	0.98 (0.52–1.83)	0.33	0.849	0.0
Cut-off					
20%	5	1.62 (0.58–4.50)	24.59	< 0.001	83.7
Others	4	1.37 (1.05–1.78)	0.70	0.872	0.0
Publication year					
≥ 2009	5	1.09(0.73–1.61)	9.18	0.057	56.4
< 2009	4	2.20(0.81–5.94)	11.14	0.011	73.1
Patient age (years)					
> 65	5	0.92(0.64–1.33)	5.45	0.245	26.6
≤ 65	4	2.43(1.16–5.12)	10.06	0.018	70.2

### Impact of p53 expression on PFS

The relationship between p53 expression and PFS in NMIBC patients treated with BCG is illustrated in Fig. 2B. The pooled HR for all 8 articles was 1.37 (95% CI: 0.90–2.09), which had significant heterogeneity (I^2^ = 44.7%, *P* = 0.081). In the stratified analyses by the region, sample size, follow-up time, stage, cut-off, publication year, and patient age, significant associations were only observed in the studies with sample sizes smaller than 100, follow-up shorter than 60 months, and patient age ≤ 65 years (HR: 1.97, 95% CI: 1.04–3.74; HR: 2.37, 95% CI: 1.23–4.55; HR: 2.49, 95% CI: 1.19–5.21, respectively) (Table 3).

**Table 3:** Subgroup results of PFS and heterogeneity test.

			Heterogeneity test		
Variables	Study number	HR (95% CI)	Q	*P*	I^2^ (%)
Total PFS	8	1.37 (0.90–2.09)	12.65	0.081	44.7
Region					
Asian	2	3.13 (0.83–11.80)	0.15	0.703	0.0
Caucasian	6	1.26 (0.81–1.95)	9.84	0.080	49.2
Sample size					
≥ 100	2	0.78 (0.43–1.42)	0.22	0.637	0.0
< 100	6	1.97 (1.04–3.74)	11.52	0.042	56.6
Follow-up (months)					
≥ 60	5	1.10 (0.70–1.72)	6.47	0.166	38.2
< 60	3	2.37 (1.23–4.55)	0.09	0.956	0.0
Stage					
All NMIBC	3	1.58 (0.60–4.12)	3.61	0.164	44.7
T1G3	4	1.86 (0.80–4.34)	6.11	0.106	50.9
Cut-off					
20%	4	1.89 (0.84–4.25)	4.98	0.173	39.8
Others	4	1.04 (0.87–1.25)	3.20	0.362	6.1
Publication year					
≥ 2009	4	1.41(0.54–3.70)	6.46	0.091	53.6
< 2009	4	1.53(0.85–2.78)	6.18	0.103	51.4
Patient age (years)					
> 65	6	1.13(0.76–1.68)	7.23	0.204	30.8
≤ 65	2	2.49(1.19–5.21)	0.00	0.984	0.0

### Galbraith plot and meta-regression

**Fig 3:**
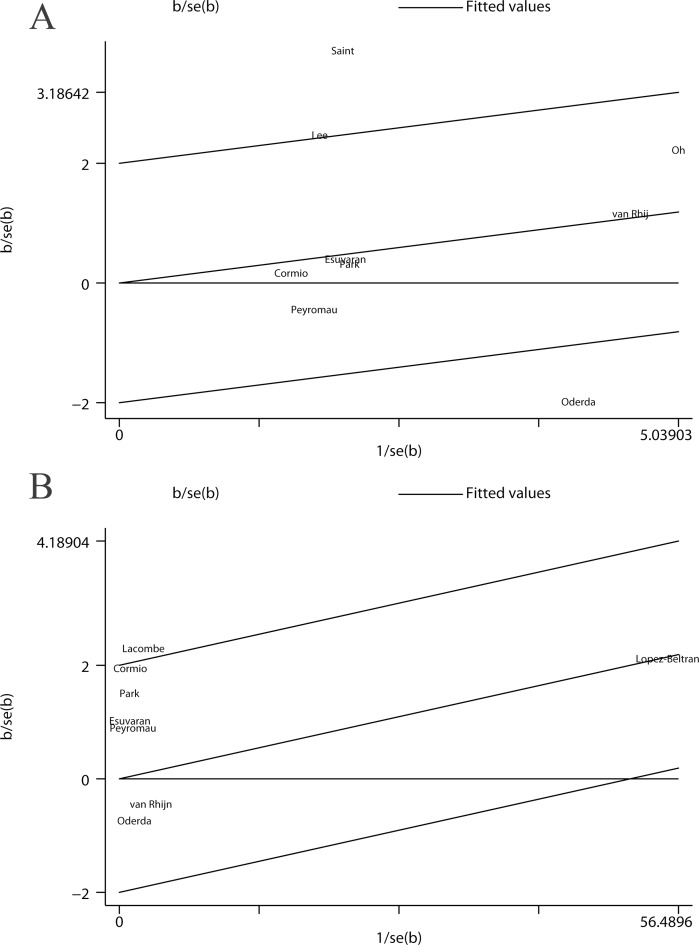
Galbraith plot analysis was used to evaluate heterogeneity. It suggested that two studies were the potential source of heterogeneity for RFS (A), while one for PFS (B).

Through the Galbraith plot, the studies performed by Saint et al. [12] and Oderda et al. [15] were detected as the major sources of heterogeneity for RFS (Fig. 3A). After excluding these 2 studies, there was no evidence of heterogeneity across the remaining studies (I^2^ = 0.0%, *P* = 0.499) and the overall association became statistically significant (HR: 1.38, 95% CI: 1.08–1.77). Furthermore, the Galbraith plot suggested that the study conducted by Lacombe et al. [17] contributed to the heterogeneity for PFS (Fig. 3B). After excluding this study, the pooled HR for the remaining 7 studies was 1.15 (95% CI: 0.79–1.68) and heterogeneity was obviously reduced (I^2^ = 25.7%, *P* = 0.233). Furthermore, seven factors (publication year, region, sample size, stage, cut-off, patient age, and follow-up time), which may contribute to the heterogeneity, were assessed by meta-regression. As a result, only the patient age was identified as a possible source of heterogeneity for RFS (*P* = 0.05).

### Cumulative meta-analysis

Cumulative meta-analysis, in the order of the publication time, was conducted. S1 Fig. shows the results from the cumulative meta-analysis of the association between p53 and RFS or PFS, which are given in chronologic order. The 95% confidence intervals gradually narrowed with the increasing number of included studies, suggesting that the precision of the summary estimates was progressively boosted by continually enrolling more cases.

### Publication bias

**Fig 4:**
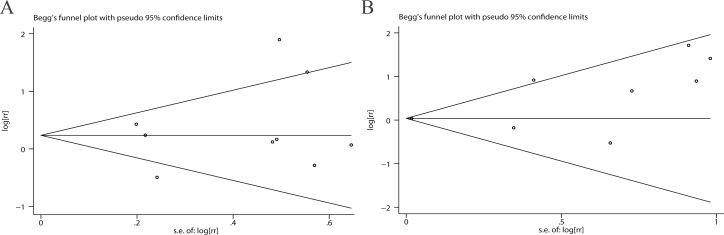
Funnel plots of p53 expression and RFS (A) or PFS (B).

In the present meta-analysis, using Begg’s and Egger’s tests, there was no evidence of significant publication bias among the studies with respect to RFS (*P* = 0.754, 0.488) or PFS (*P* = 0.174, 0.110) (Fig. 4). In addition, the trim-and-fill method suggested that there was no significant change after trimming and filling (data not shown).

## Discussion

The current meta-analysis summarizes the results of the published data, which are based on a total of 11 studies (including over 1,000 cancer cases). To the best of our knowledge, this is the first meta-analysis to evaluate the relationship between p53 status and the prognosis of NMIBC patients treated with BCG. The results suggested that overall there was no clear relationship between them. However, in stratified analysis by region, overexpression of p53 was a predictor of poor RFS in Asian populations. This finding suggested that the ethnicity or geographic settings might play an important role in the impact of p53 overexpression on the prognosis of NMIBC patients who undergo BCG treatment after initial TURB.

The Galbraith plot suggested that the observed heterogeneity among the studies of p53 status and RFS could be explained by the studies conducted by Saint et al. [12] and Oderda et al. [15]. These studies reported the strongest positive and negative relationships (HR: 6.67, 95% CI: 2.38–16.67; HR: 0.61, 95% CI: 0.38–0.98, respectively). After excluding these 2 studies, the association between p53 overexpression and RFS in NMIBC patients treated with BCG became significant (HR: 1.38, 95% CI: 1.08–1.77) without any evidence of study heterogeneity (I^2^ = 0.0%, *P* = 0.499). These results suggested that the heterogeneity across the included studies might have led to an underestimation of the risk estimate for RFS, and p53 was likely to be a potential predictor of RFS.

The prognosis of NMIBC, also called superficial bladder cancer, is heterogeneous and clinical treatments vary considerably between patients [31–33]. According to the recommendations of the 2013 European Association of Urology (EAU) guidelines, the risks of both recurrence and progression could be estimated for individual patients by applying the European Organization for Research and Treatment of Cancer (EORTC) scoring system, which incorporates the following six most important clinical and pathologic factors: tumor number, grade, size, prior recurrence rate, T category, and concomitant carcinoma in situ (CIS). Using the above characteristics, patients can be divided into several risk groups with different adjuvant therapy, either chemotherapy or immunotherapy with BCG [34]. In addition, the Spanish Urological Club for Oncological Treatment (CUETO) scoring model was developed for BCG-treated patients to predict the short- and long-term probabilities of recurrence and progression and the discrimination was superior to EORTC in BCG-treated patients [34,35]. However, accurately predicting which NMIBC patients will experience recurrence or progression to muscle-invasive disease is still very difficult [36]. Therefore, there is a need for new and reliable prognostic factors.

In human cancers, p53 is the most commonly inactivated tumor suppressor gene [37]. Loss of expression of wild-type p53 contributes to abnormal cell proliferation and tumorigenesis, while mutant p53 can gain new abilities promoting cell migration, invasion and metastasis, which are partially from interference with p63 function [38]. Many IHC studies have confirmed that p53 is highly overexpressed in bladder cancer patients and that it is associated with advanced bladder tumor stage and grade [39,40]. Furthermore, p53 overexpression appears to correlate with the mitotic index and vascular invasion [41]. Therefore, p53 is a potential surrogate biomarker for NMIBC patients who are treated with BCG. As mentioned previously, several studies have investigated the influence of p53 overexpression on NMIBC patients who have undergone intravesical BCG treatment after initial TURB. However, the results of these reports are still controversial. As individual studies may have insufficient statistical power, our meta-analysis of 11 studies, involving a great many NMIBC patients, enhanced the statistical power and provided more reliable estimates.

To date, various meta-analyses have evaluated the relationship between p53 and bladder cancer with a focus on other topics. For example, several meta-analyses have assessed the relationship between p53 Arg72Pro polymorphism and bladder cancer risk [42–46]. As a consequence, most of these reported that p53 Arg72Pro polymorphism was associated with an increased risk of bladder cancer in Asians but not in Caucasians [42–45]. This result was similar to our study, which also indicated that the association was more robust in Asians than Caucasians. Therefore, p53 may really have race-specific effects on bladder cancer. With respect to the prognosis, the meta-analysis conducted by Malats et al, which was published in 2005, suggested that the evidence was not sufficient to conclude whether p53 could serve as a marker of the outcome in patients with bladder cancer [47].

Several important limitations should be considered in interpreting the results of our meta-analysis. First, this meta-analysis was limited by the presence of heterogeneity across the studies. The heterogeneity may be from the differences in the characteristics of the patients, technical platforms, cut-off values, and follow-up time. Second, although the Begg’s and Egger’s test did not indicate any evidence of publication bias, the results may have been influenced by publication bias, because only studies published in English and Chinese were searched and included. Finally, there was a wide range of values for the cut-off points for the low and high categories of p53 expression in the included studies, which might also have an impact on the current analysis. Therefore, large cohort studies must be performed in the future with uniform criteria for high p53 expression.

In conclusion, our study suggests that, based on the available information, p53 overexpression in NMIBC patients treated with BCG may be associated with RFS, especially in Asian populations. Because of the limitations discussed above, further studies with rigid criteria and large populations are still warranted to confirm the findings of our study.

## Supporting Information

S1 PRISMA Checklist (DOC)

S1 Fig Results from cumulative meta-analysis of the association between p53 expression and RFS (A) or PFS (B). (TIF)
