# Supplementary figures and images for: p53 Status Correlates with the Risk of Recurrence in Non-Muscle Invasive Bladder Cancers Treated with Bacillus Calmette–Guérin: A Meta-Analysis
Source: PLoS One. 2015 Mar 5;10(3):e0119476. doi: 10.1371/journal.pone.0119476 (PMC4351250; doi:10.1371/journal.pone.0119476)

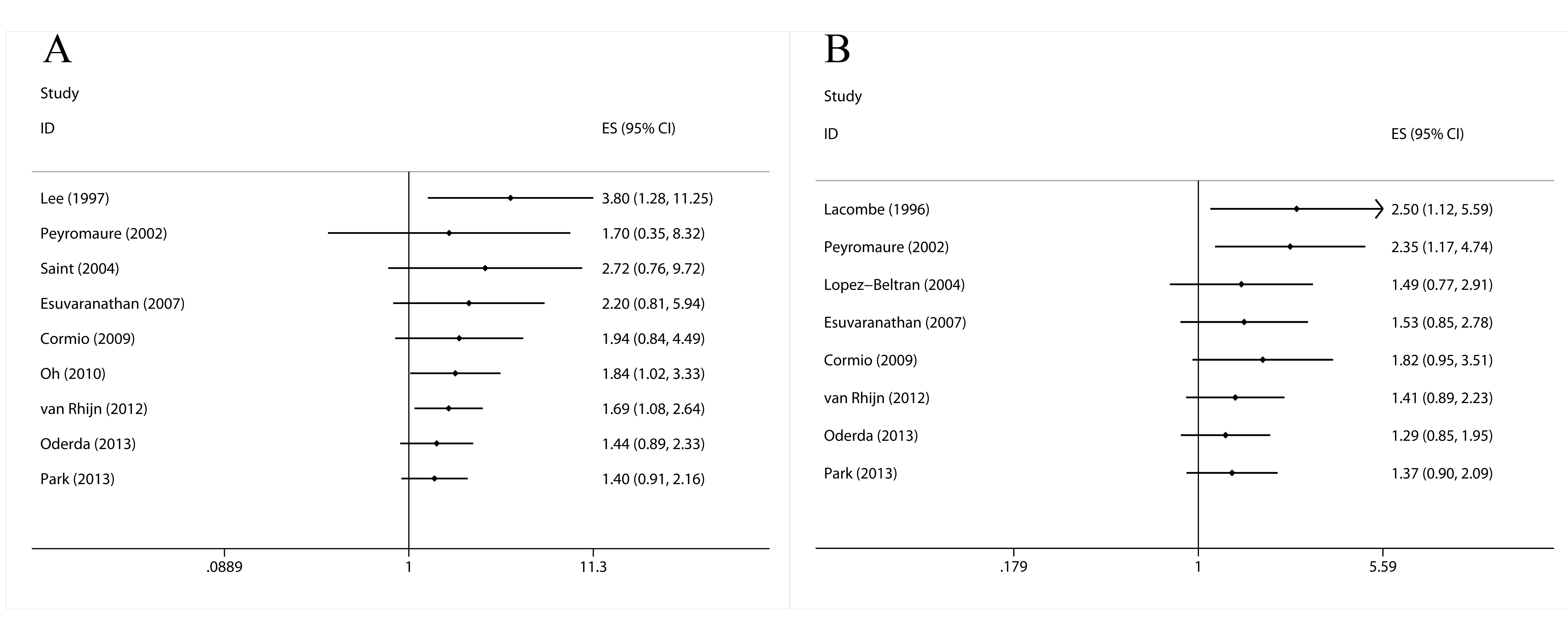

Supplement: S1 Fig — (TIF) [file pone.0119476.s002.tif]
